# PD-L1 Status in Tenosynovial Giant Cell Tumors

**DOI:** 10.3390/medicina58091270

**Published:** 2022-09-13

**Authors:** Tulay Zenginkinet, Abdullahi Umar Faruq, Ayse Nur Toksoz Yildirim, Yusuf Iyetin, Burak Ozturan, Erhan Okay, Aykut Celik, Korhan Ozkan, Muhlik Akyurek

**Affiliations:** 1 Department of Pathology, Istanbul Medeniyet University Prof. Dr. Suleyman Yalcin City Hospital, 34730 Istanbul, Turkey; 2 Department of Trauma & Orthopaedic Surgery, General Amadi Rimi Specialist Hospital, Katsina 820101, Nigeria; 3 Department of Orthopedics and Traumatology, Istanbul Pendik Bolge Hospital, 34890 Istanbul, Turkey; 4 Department of Orthopaedics and Traumatology, Istanbul Medeniyet University, Goztepe Training and Research Hospital, 34730 Istanbul, Turkey; 5 Department of Orthopaedic and Traumatology, Maria Josef Hospital, 48268 Greven, Germany

**Keywords:** tenosynovial giant cell tumor, PD-1/PD-L1, immunohistochemistry

## Abstract

*Background and Objectives*: Tenosynovial giant cell tumors (TSGCTs) are benign soft tissue tumors that are divided into localized- and diffuse-type tumors, according to the World Health Organization classification of soft tissue tumours. The diffuse-type TSGCT sometimes behave aggressively and poses treatment challenges especially in patients with neurovascular involvement. Symptomatic patients who are not good candidates for surgery due to high morbidity risk may benefit from medical therapy. *Objectives*: Drugs that target programmed death ligand 1 (PD-L1) are among a new generation of medical therapy options, which, recently, have been explored and have displayed promising results in various cancer types; therefore, we aimed to investigate the PD-L1 status of TSGCTs as a possible therapeutic target. *Materials and Methods*: We assessed the PD-L1 status of 20 patients (15 men and 5 women, median age = 39 years) that had been diagnosed with TSGCTs in a single institution, between 2018 and 2020. The patients had localized- (*n* = 7) and diffuse-type (*n* = 13) TSGCTs. Formalin-fixed paraffin-embedded (FFPE) blocks were retrospectively retrieved from the pathology department. An immunohistochemical analysis was performed in sections of 3 micron thickness from these blocks. *Results*: Seventy-five percent of our patients with TSGCTs were immunopositive to PD-L1 staining. *Conclusions*: Taking into consideration the high positivity rate of PD-L1 staining in TSGCTs, PD-L1 blockage may be used as a valuable medical treatment for TSGCTs; however, further studies are needed.

## 1. Introduction

Tenosynovial giant cell tumors (TSGCTs) are rare benign tumors. According to the World Health Organization (WHO) classification, there are two subtypes of TSGCTs, i.e., localized and diffuse. Although they are benign, the diffuse-type TSGCTs are aggressive and may cause significant morbidity [1,2,3]. They usually originate from joint capsules, bursae, and tendon sheaths. In the majority of cases, a specific chromosomal translocation involving t(1;2) (CSF-1 and COL 6A3) results in overexpression of colony stimulating factor 1 (CSF-1) that results in recruitment of CSF-1 receptor positive macrophages, giant cells, and osteoclasts [4]. According to the Danish 2017 registry, the prevalence of localized-type TSGCTs is 44/100,000, whereas it is 12/100,000 for diffuse-type TSGCTs [5]. Although exceptionally rare, malignant transformation may occur, which usually consists of a coexisting benign TSGCT juxtaposed with a sarcoma and is called a primary malignant TSGCT or sarcomatous recurrence of a previously diagnosed benign TSGCT (secondary malignant TSGCT) [6,7]. Diffuse-type TSGCTs and recurrences may involve neurovascular structures and may pose diffuculties in treatment strategies. Surgery may cause great morbidity and may even lead to amputation in rare cases. Therefore, there is still no consesus regarding treatment of, especially, some diffuse and recurrent diseases [8]. PD-L1 (Programmed Cell Death Ligand 1) is the ligand for PD-1 (Programmed Death-1) and is presented on T cells, B cells, macrophages, and dendritic cells. Activation of the PD-1/PD-L1 complex reduces antigen-specific T cell activity, and decreases the number of antigen-specific T cells by inducing apoptosis. There are many studies that have shown increased expression of PD-L1 ligand in various cancers originating from the lungs, skin, ovaries, cervix, esophagus, breasts, bladder, brain, bone, kidneys, and liver. However, there is only one study published in English in the literature regarding PD-L1 expression in TSGCTs [8,9].

Therefore, in this study, we aimed to investigate the programmed death ligand 1 (PD-L1) status of TSGCTs in order to look for a possible therapeutic usage of PD-L1 blockage.

## 2. Materials and Methods

Between 2018 and 2020, 20 patients who presented to the orthopedic oncology clinic of stanbul Medeniyet University Prof. Dr. Suleyman Yalcin City Hospital and who were diagnosed with TSGCTs in resection materials were included in this study. The patients were informed about the study. Ethical approval was obtained from the editorial board of the institution (approval number 2021/0438, 25 August 2021).

Pathological slides which were not technically feasible for immunohistochemical analysis, patients with incomplete patient data, patients who did not undergo resection, and those whose blocks could not be obtained were not included in the study. Patients who had at least one year of follow up and had last control in the last six months were included in the study. Clinico-pathological characteristics such as gender, age, and follow-up data (recurrence and outcome) were retrospectively collected from patients’ medical records. Tissue samples obtained from operation specimens were used to prepare formalin-fixed paraffin-embedded (FFPE) blocks. Sections of 3 micron thickness were taken from these blocks for immunohistochemical analysis. Slides were stained for PD-L1 antibody (Cell Signaling, E1L3N) using Leica Bond Autostainer. Placenta and tonsil tissues were used as an external control and lymphocytes were used as an internal control.

We categorized the strength of positivity of samples as follows: from 1% to 4% positive cells as very weak (Grade 1), from 5% to 9% positive cells as weak (Grade 2), from 10% to 49% positive cells as moderate (Grade 3), and greater than 50% positive cells as strong expression (Grade 4) [10,11]. PD-L1 expression was classified as positive if a distinct membranous or cytoplasmic staining with 5% or greater expression was observed; therefore, Grade 1 was not taken into account as they were weakly stained, as recommended by Zeng et al. [12]. All slides were reviewed by two experienced pathologists. A chi-square test was used to determine association between TSGCT type (diffuse/localized) and PD-L1 staining grade among 15 PD-L1 positive cases. The significance level, in this study, was set at (*p*-value < 0.05). A chi-square test was also used to determine the association between tumor size and PD-L1 positivity which involved Grade 2-3-4 immunostaining or PD-L1 negativity which involved Grade 1 immunostaining in our study. The significance level in this study was again set at (*p*-value < 0.05). A Mann–Whitney U Test was used to determine the association between age and PD-L1 positivity or negativity.

## 3. Results

Clinico-pathological characteristics such as gender, age, recurrence and outcome were also presented. Seven patients had localized-type TSGCTs and 13 patients had diffuse-type TSGCTs. There were only 2 recurrences, one from a localized-type TSGCT, and one from diffuse-type TSGCT (Table 1).

**Figure 1:**
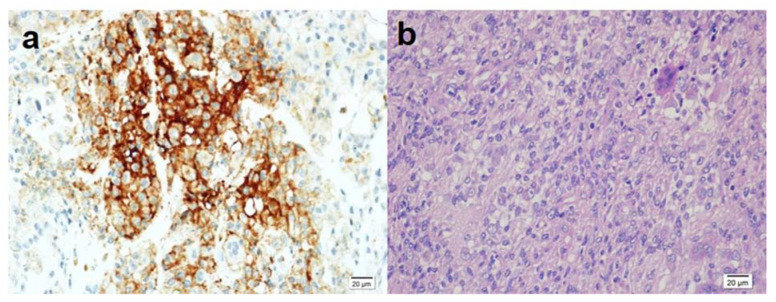
(**a**) Positive expression of PD-L1 in mononuclear cells, 400× magnification; (**b**) H&E staining of the same sectional area, 400× magnification.

**Figure 2:**
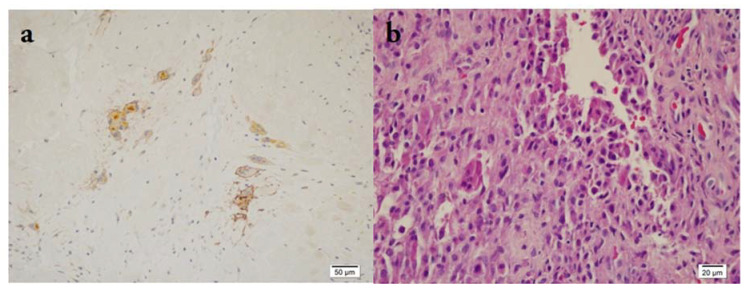
(**a**) Positive expression of PD-L1 in multinuclear cells, 200× magnification; (**b**) H&E staining of the same sectional area, 400× magnification.

All our specimens were positively stained with PD-L1 immunohistochemical staining. However, if the cut-off value for immunopositivity was accepted as 5% according to a study by Zheng et al. [12], then positive staining was observed in 14 of the total 20 patients (70%). Five patients were stained as Grade 1, six patients as Grade 2, seven patients as Grade 3, and two patients as Grade 4. Staining properties of mononuclear cells, multinucleated cells, and foam cells were noted (Figure 1 and Figure 2). There was no statistically significant relationship between type (localized or diffuse) and PD-L1 immunohistostaining (Table 2). There were only two recurrences, one localized-type tumor and one diffuse-type tumor with Grade 3 positivity for PD-L1 immunohistostaining. There was no statistically significant relationship between PD-L1 positivity or negativity and tumor size (*p* > 0.05) (Table 3). There was also no statistically significant relationship between PD-L1 positivity or negativity and the age of patients (*p* = 0.826 and *p* > 0.01)) (Table 4).

## 4. Discussion

A tenosynovial giant cell tumor is a proliferative and inflammatory type of tumor that originates from the synovium of joints, bursae, and tendon sheaths [11]. Jaffe et al., in 1941, used the term pigmented villonodular synovitis, bursitis, and tenosynovitis for these lesions [12,13] with a slight female dominance, as in our study. 

Localized-type tumors usually affect patients in the fourth and fifth decades and usually occur around the digits (85%), while diffuse-type tumors predominately occur within major joints, and 75% of cases are in the knee joint, with a propensity for patients less than 40 years old [14].

In this study, three out of the seven localized-type tumors were detected in the digits and six of the nine tumors in knees had been affected with diffuse-type tumors.

Intra-articular forms create discomfort with repeated swelling and bleeding into the joint, thus, causing restricted range of motion and joint degeneration in the long term [15,16]. Marginal excision for localized-type and total synovectomy for diffuse-type TSGCTs is generally preferred whenever feasible [17,18]. Recurrence risk increases with incomplete resection, bone and muscle, tendon, or neurovascular involvement [19,20]. It is usually impossible to have microscopically negative margins in diffuse-type TSGCTs. Therefore, multiple operations in some patients are inevitable due to recurrence which increases morbidity. In addition, scar tissues around the major neurovascular structures make surgery even more challenging [21,22]. External beam irradiation is another treatment option. It has been used mostly in inoperable cases and persistent recurrent disease; however, sarcomatous transformation and pathological fractures are major possible complications [20,23].

Although very rare, tumors may undergo malignant transformation. Prognosis for malignant TSGCTs is extremely poor with common lymph node and lung metastases. The mean survival is only 22.5 months [24].

Microscopically tenosinovial giant cell tumors have a lobular architecture with fibrous bands traversing the lesion. They consist of large histiocytoid cells with eosinophilic cytoplasm and eccentrically placed nuclei, foamy histiocytes, mononuclear stromal cells, hemosiderin-laden histiocytes, and osteoclast-like giant cells [15,16,17].

West et al. demonstrated that translocation, involving the 1p13 locus of some mononuclear and multinuclear cells (2–16% of the cell population), caused hyperexpression of CSF-1 that eventually caused recruitment of macrophages bearing CSFR-1 receptor which, in turn, differentiated into multinuclear cells and created the so-called multinuclear landscape of a TSGCT [25].

All patients with TSGCTs display elevated levels of CSF-1. A Phase 1 study using intravenous emactuzumab (anti-CSF1R monoclonal antibody), in patients with locally advanced diffuse-type TSGCTs, reported 86% objective response rate with 7% complete response and 79% partial response. The most common adverse effects were facial edema, asthenia, and pruritus [26]. A Phase 2 study of twice daily oral nilotinib reported a 90% rate of stable disease with no complete or partial response in patients with inoperable or relapsing PVNS or PVNS only resectable with mutilating surgery. However, 41% of patients required treatment modification due to adverse effects [27]. There have been ongoing studies, especially in recent years, on the medical treatment of TSGCTs in selected patients whose surgery would result in major morbidity [28]. CSF-1R inhibitor tyrosine kinase pexidartinib is the first approved systemic therapy for patients with tenosynovial giant cell tumors; however, it may cause potentially life-threatening mixed or cholestatic hepatotoxicity [29]. Imatinib mesylate (IM) blocks the CSF-1 receptor and it has also been associated with one- and five-year progression-free survival rates of 71% and 48%, respectively. The most common adverse effects were fatigue, edema, and nausea [30].

Currently, there is an increasing focus on programmed cell death protein (PD-1) and its ligand (PD-L1) as an area of clinical interest for cancer treatment. Inhibition of the PD-1/PD-L1 axis has hindered proliferation and induced remission in various human cancers.

Programmed cell death protein 1 (PD-1) and programmed cell death ligand 1 (PD-L1) were discovered in 1992 and have been shown to have an important role in cancer immune escape [31]. The balance of positive and negative signals are as important as the activation of cytotoxic T lymphocytes for the development of antitumor immunity. Negative signals are usually generated by surface molecules such as cytotoxic T lymphocyte antigen 4 (CTLA-4) and PD-1. PD-1 is an inhibitory receptor that is a member of the CD28 family and has an important role in tumor immune escape. Recently, anti-PD-L1 treatment has been investigated in various cancer types [32,33]. PD-L1 is the ligand for PD-1 and is presented on T cells, B cells, macrophages, and dendritic cells. Activation of the PD-1/PD-L1 complex reduces antigen-specific T cell activity and decreases the number of antigen-specific T cells by inducing apoptosis. There are many studies that have shown increased expression of PD-L1 ligand in various cancers originating from the lungs, skin, ovary, cervix, esophagus, breasts, bladder, brain, bone, kidneys, and liver [34,35,36,37,38,39].

According to Zheng et al., PD-L1 was positively expressed in 52.5% of all patients (21 out of 40 patients) which has been the only study about the PD-L1 status of TSGCTs [12]. Among the patients, mononuclear cells, multinucleated cells, and foam cells with positive PD-L1 expression were observed in 52.5%, 25.0%, and 17.5% of the patients, respectively. As compared with their study, in our study, 75% of all samples displayed PD-L1 expression and, among them, mononuclear cells, multinucleated cells, and foam cells with positive PD-L1 expression were observed in 20%, 70%, and 30% of the patients, respectively.

A tenosinovial giant cell tumor is caused by translocation involving the 1p13 locus comprimising a subset of lesional cells that cause hyperexpression of CSF-1 resulting in recruitment of mononuclear and multinucleated giant cells with other mixed-type inflammatory cells. Therefore, PD-L1 positivity of both mononuclear and multinuclear cells is important.

In our study, there were only two recurrences, one localized-type tumor and one diffuse-type tumor, with Grade 3 positivity for PD-L1 immunohistostaining. Therefore, it was not possible to draw a conclusion based upon two cases, but PD-L1 positivity might have a role in recurrence. Although Zheng et al. [12] found a statistically positive correlation between PD-L1 positivity and tumor size, no statistically significant difference was found as comparing with tumor size and PD-L1 immunopositivity in our study, which might be due to positive expression of PD-L1 in 75% of all patients as compared with 52.5% expression according to the study by Zheng et al.

Inhibition of PD-1/PD-L1 has hindered proliferation and introduced remission in various human cancer types; therefore, there has been a new increased focus on PD-1/PD-L1 blockage in many tumoral cases. Based on our results, further evolution of medical treatment for PD-L1 blockage is warranted.

## 5. Conclusions

Despite improvements and developments in medical therapy, research is still being conducted to find the best possible treatment with the highest efficacy and fewest side effects. A new generation of drugs that target PD-1/PD-L1 display promising results, especially, in recent studies for various cancer types. According to our results, it may be a valuable therepeutic option for TSGCT treatment, in which surgery may pose significant challenges and morbidity, and may even lead to loss of an extremity. However, more studies are needed before a definitive decision is reached.

## Figures and Tables

**Table 1:** Tumor characteristics.

S/N	Patient	Age (Years)	Sex	Tumor Type (Diffuse/Localized)	Tumor Size (cm)	Recurrence	Location	PD-L1 Immunostaining	Multinucleated Cells M	Mononuclear Cells	Foam Cells
1.	H.S.	42	F	Diffuse	8.0 × 5.0 × 4.0	None	Knee	52% (4)	+	+	+
2.	E.T.	38	M	Diffuse	16 × 10 × 3.5	None	Hip	3% (1)	+	−	−
3.	S.S.	32	F	Localized	2.5 × 1.2 × 1.2	None	Knee	6% (2)	+	−	−
4.	T.E.	55	M	Diffuse	2.5 × 2.0 × 0.5	None	Ankle	1% (1)	+	−	−
5.	S.S.	14	M	Diffuse	3.0 × 2.0 × 0.5	None	Hip	5% (2)	+	−	−
6.	E.T.S.	45	F	Localized	2.0 × 1.5 × 1.0	None	Finger	45% (3)	+	−	−
7.	D.S.	38	F	Diffuse	13 × 4.0 × 1.5	Once	Knee	12% (3)	+	−	+
8.	N.Y.	56	M	Diffuse	3.0 × 2.2 × 1.0	None	Hip	4% (1)	+	+	−
9.	H.C.B.	16	M	Diffuse	10 × 8.0 × 2.0	None	Knee	4% (1)	+	−	−
10.	R.K.	70	F	Diffuse	1.5 × 1.5 × 0.3	None	Finger	40% (3)	+	−	+
11.	G.Y.	38	F	Localized	2.0 × 1.5 × 0.5	None	Knee	5% (2)	+	−	−
12.	L.S.	45	F	Localized	2.0 × 1.0 × 0.8	None	Finger	9% (2)	+	−	−
13.	F.K.	39	F	Diffuse	10 × 9.0 × 1.0	None	Knee	30% (3)	+	+	+
14.	S.A.	40	F	Diffuse	1.0 × 0.2 × 2.0	None	Knee	6% (2)	+	−	−
15.	V.E.	39	F	Diffuse	7.0 × 3.0 × 1.5	None	Knee	1% (1)	−	+	−
16.	M.E	64	F	Localized	3.0 × 2.5 × 2.5	None	Leg	8% (2)	−	+	−
17.	S. G	17	F	Diffuse	5.0 × 4.5 × 2.5	None	Ankle	10% (3)	+	−	−
18	A.Z.	38	F	Localized	1.5 × 1.3 × 0.3	None	Knee	50% (4)	+	+	+
19.	E.C.	31	F	Diffuse	4.5 × 4.1	None	Ankle	30% (3)	+	−	+
20.	F.M	48	F	Localized	1.0 × 0.5 × 0.5	Twice	Finger	40% (3)	+	−	−

**Table 2:** Tumor type (localized or diffuse) and PD-L1 staining.

	Percentage of Staining	Type	
		Localized	Diffuse
PD-L1 immunohistochemical staining	5–9%	4 (66.7%)	2 (33.3%)
	10–49%	2 (28.6%)	5 (71.4%)
	≥50%	1 (50%)	1 (50%)

Chi-square test: *p* = <0.01.

**Table 3:** Tumor size and grade.

		PD-L1 Immunostaining		*p*
		Grade 1	Grade 2-3-4	
Tumor size	<5	1 (20%)	9 (60%)	0.152
	>5	4 (80%)	6 (40%)	

Chi-Square Test: *p* < 0.01.

**Table 4:** Tumor grade and age.

		*n*	Average ± Ss	Min-Max (Median)	*p*
Age	Grade 1	5	40.8 ± 16.27	16–56 (39)	0.826
	Grade 2-3-4	15	40.07 ± 14.53	14–70 (39)	

Mann–Whitney U Test: *p* < 0.05, *p* < 0.01.

## Data Availability

The study did not report any data.

## References

[1] Lucas D.R. (2012). Tenosynovial giant cell tumor: Case report and review. Arch. Pathol. Lab. Med..

[2] De Saint Aubain Somerhausen N., van de Rijn M., Fletcher C.D.M., Bridge J.A., Hogendoorn P.C.W., Mertens F. (2013). Tenosynovial giant cell tumour. WHO Classification of Tumours of Soft-Tissue and Bone.

[3] Gouin F., Noailles T. (2017). Localized and diffuse forms of tenosynovial giant cell tumor (formerly giant cell tumor of the tendon sheath and pigmented villonodular synovitis). Orthop. Traumatol. Surg. Res..

[4] Peyraud F., Cousin S., Italiano A. (2017). CSF-1R Inhibitor Development: Current Clinical Status. Curr. Oncol. Rep..

[5] Ehrenstein V., Andersen S.L., Qazi I., Sankar N., Pedersen A.B., Sikorski R., Acquavella J.F. (2017). Tenosynovial Giant Cell Tumor: Incidence, Prevalence, Patient Characteristics, and Recurrence. A Registry-based Cohort Study in Denmark. J. Rheumatol..

[6] Imakiire N., Fujino T., Morii T., Honya K., Mochizuki K., Satomi K., Fujioka Y. (2011). Malignant pigmented villonodular synovitis in the knee—Report of a case with rapid clinical progression. Open Orthop. J..

[7] Theunissen C.I.J.M., Bras J., van Lienden K.P., Obdeijn M.C. (2013). Malignant giant cell tumor in the carpal tunnel: A case report and review of literature. J. Wrist Surg..

[8] Latchman Y., Wood C.R., Chernova T., Chaudhary D., Borde M., Chernova I., Iwai Y., Long A.J., Brown J.A., Nunes R. (2001). PD-L2 is a second ligand for PD-1 and inhibits T cell activation. Nat. Immunol..

[9] Ravetch J.V., Lanier L.L. (2000). Immune inhibitory receptors. Science.

[10] Healey J.H., Bernthal N.M., van de Sande M. (2020). Management of Tenosynovial Giant Cell Tumor: A Neoplastic and Inflammatory Disease. JAAOS Glob. Res. Rev..

[11] Wang X., Teng F., Kong L., Yu J. (2016). PD-L1 expression in human cancers and its association with clinical outcomes. OncoTargets Ther..

[12] Zheng B., Yu L., Hu J., Xu H., Wang J., Shi Y., Luo X., Yan W. (2019). Expression of PD-L1 in mononuclear cells, multinucleated cells, and foam cells in tenosynovial giant cell tumors. Int. J. Clin. Exp. Pathol..

[13] Jaffe H.L., Lichtenstein L., Sutro C.J. (1941). Pigmented villonodular synovitis, bursitis and tenosynovitis. Arch. Pathol..

[14] Yang C.-Y., Lin M.-W., Chang Y.-L., Wu C.-T., Yang P.-C. (2014). Programmed cell death-ligand 1 expression in surgically resected stage I pulmonary adenocarcinoma and its correlation with driver mutations and clinical outcomes. Eur. J. Cancer.

[15] Weiss S.W., Goldblum J.R. (2008). Benign tumors and tumor-like lesions of synovial tissue. Enzinger & Weiss’s Soft Tissue Tumors.

[16] Adams E.L., Yoder E.M., Kasdan M.L. (2012). Giant cell tumor of the tendon sheath: Experience with 65 cases. Eplasty.

[17] Ushijima M., Hashimoto H., Tsuneyoshi M., Enjoji M. (1986). Giant cell tumor of the tendon sheath (nodular tenosynovitis). A study of 207 cases to compare the large joint group with the common digit group. Cancer.

[18] Boland J.M., Folpe A.L., Hornick J.L., Grogg K.L. (2009). Clusterin is expressed in normal synoviocytes and in tenosynovial giant cell tumors of localized and diffuse types: Diagnostic and histogenetic implications. Am. J. Surg. Pathol..

[19] Kuhnen C., Müller K.M., Rabstein S., Kasprzynski A., Herter P. (2005). Tenosynovialer Riesenzelltumor Morphologische, ultrastrukturelle und immunhistochemische Befunde sowie Differenzia ldiagnose riesenzellhaltiger Tumoren des Weichgewebes [Tenosynovial giant cell tumor]. Pathologe.

[20] Somerhausen N.S., Fletcher C.D. (2000). Diffuse-type giant cell tumor: Clinicopathologic and immunohistochemical analysis of 50 cases with extraarticular disease. Am. J. Surg. Pathol..

[21] Botez P., Sirbu P.D., Grierosu C., Mihailescu D., Savin L., Scarlat M.M. (2013). Adult multifocal pigmented villonodular synovitis—Clinical review. Int. Orthop..

[22] Dines J.S., DeBerardino T., Wells J.L., Dodson C.C., Shindle M., DiCarlo E.F., Warren R.F. (2007). Long-term follow-up of surgically treated localized pigmented villonodular synovitis of the knee. Arthrosc. J. Arthrosc. Relat. Surg..

[23] Palmerini E., Staals E.L., Maki R.G., Pengo S., Cioffi A., Gambarotti M., Picci P., Daolio P.A., Parafioriti A., Morris C. (2014). Tenosynovial giant cell tumour/pigmented villonodular synovitis: Outcome of 294 patients before the era of kinase inhibitors. Eur. J. Cancer.

[24] Martin R.C., Osborne D.L., Edwards M.J., Wrightson W., McMasters K.M. (2000). Giant cell tumor of tendon sheath, tenosynovial giant cell tumor, and pigmented villonodular synovitis: Defining the presentation, surgical therapy and recurrence. Oncol. Rep..

[25] Tsukamoto S., Zucchini R., Staals E., Mavrogenis A.F., Akahane M., Palmerini E., Errani C., Tanaka Y. (2020). Incomplete resection increases the risk of local recurrence and negatively affects functional outcome in patients with tenosynovial giant cell tumor of the hindfoot. Foot Ankle Surg..

[26] Kotwal P.P., Gupta V., Malhotra R. (2000). Giant-cell tumour of the tendon sheath. Is radiotherapy indicated to prevent recurrence after surgery?. J. Bone Jt. Surg..

[27] Shi J., Zheng J., Zhou X., Li Z., Chen X., Gao W., Yan H. (2019). Risk Factors Associated With Postoperative Recurrence in Patients With Tenosynovial Giant Cell Tumor of the Hand: A Retrospective Cohort Study. Ann. Plast. Surg..

[28] Mastboom M.J.L., Palmerini E., Verspoor F.G.M., Rueten-Budde A.J., Stacchiotti S., Staals E.L., Schaap G.R., Jutte P.C., Aston W., Gelderblom H. (2019). Surgical outcomes of patients with diffuse-type tenosynovial giant-cell tumours: An international, retrospective, cohort study. Lancet Oncol..

[29] Msc A.M.G., Ferguson P.C., Catton C.N., Chung P.W.M., White L.M., Wunder J.S., Bell R.S., O’Sullivan B. (2012). Long-term outcome of the treatment of high-risk tenosynovial giant cell tumor/pigmented villonodular synovitis with radiotherapy and surgery. Cancer.

[30] Al-Ibraheemi A., Ahrens W.A., Fritchie K., Dong J., Oliveira A.M., Balzer B., Folpe A.L. (2019). Malignant Tenosynovial Giant Cell Tumor: The True “Synovial Sarcoma?” A Clinicopathologic, Immunohistochemical, and Molecular Cytogenetic Study of 10 Cases, Supporting Origin from Synoviocytes. Mod. Pathol..

[31] Han Y., Liu D., Li L. (2020). PD-1/PD-L1 pathway: Current researches in cancer. Am. J. Cancer Res..

[32] West R.B., Rubin B.P., Miller M.A., Subramanian S., Kaygusuz G., Montgomery K., Zhu S., Marinelli R.J., De Luca A., Downs-Kelly E. (2006). A landscape effect in tenosynovial giant-cell tumor from activation of CSF1 expression by a translocation in a minority of tumor cells. Proc. Natl. Acad. Sci. USA.

[33] Cassier A.P., Italiano A., Gomez-Roca A.C., Le Tourneau C., Toulmonde M., Cannarile A.M., Ries C., Brillouet A., Müller C., Jegg A.-M. (2015). CSF1R inhibition with emactuzumab in locally advanced diffuse-type tenosynovial giant cell tumours of the soft tissue: A dose-escalation and dose-expansion phase 1 study. Lancet Oncol..

[34] Gelderblom H., Pérol D., Chevreau C., Tattersall M.H., Stacchiotti S., Casali P.G., Cropet C., Piperno-Neumann S., Le Cesne A., Italiano A. (2013). An open-label international multicentric phase II study of nilotinib in progressive pigmented villo-nodular synovitis (PVNS) not amenable to a conservative surgical treatment. J. Clin. Oncol..

[35] Gelderblom H., de Sande M.V. (2020). Pexidartinib: First approved systemic therapy for patients with tenosynovial giant cell tumor. Future Oncol..

[36] Verspoor F.G.M., Mastboom M.J.L., Hannink G., Maki R.G., Wagner A., Bompas E., Desai J., Italiano A., Seddon B.M., Van Der Graaf W.T.A. (2019). Long-term efficacy of imatinib mesylate in patients with advanced Tenosynovial Giant Cell Tumor. Sci. Rep..

[37] Ishida Y., Agata Y., Shibahara K., Honjo T. (1992). Induced expression of PD-1, a novel member of the immunoglobulin gene superfamily, upon programmed cell death. EMBO J..

[38] De Visser K.E., Eichten A., Coussens L.M. (2006). Paradoxical roles of the immune system during cancer development. Nat. Rev. Cancer.

[39] Dong H., Zhu G., Tamada K., Chen L. (1999). B7-H1, a third member of the B7 family, co-stimulates T-cell proliferation and interleukin-10 secretion. Nat. Med..

